# Sluggish Cognitive Tempo in Pediatric Sickle Cell Disease

**DOI:** 10.3389/fneur.2022.867437

**Published:** 2022-07-07

**Authors:** Steven J. Hardy, Sydney Forman, Kristina K. Hardy, Jeffrey Schatz

**Affiliations:** ^1^Divisions of Hematology and Oncology, Children's National Hospital, Washington, DC, United States; ^2^Department of Psychiatry and Behavioral Sciences, The George Washington University School of Medicine and Health Sciences, Washington, DC, United States; ^3^Division of Neuropsychology, Children's National Hospital, Washington, DC, United States; ^4^Department of Psychology, University of South Carolina, Columbia, SC, United States

**Keywords:** sickle cell, cognitive, sluggish cognitive tempo, ADHD, attention, learning

## Abstract

**Background:**

Sickle cell disease (SCD) imparts risk for a range of neurodevelopmental and neurocognitive disorders. Sluggish cognitive tempo (SCT) is a distinct syndrome that often co-occurs with attention-deficit/hyperactivity disorder (ADHD) but has not been described in SCD. We investigated the reliability and validity of a SCT measure in SCD and examined associations with biopsychosocial risk factors and functional outcomes.

**Materials and Methods:**

Caregivers (*n* = 85) of children with SCD ages 7-16 reported on socio-demographics and the Kiddie-Sluggish Cognitive Tempo (K-SCT) measure, Behavior Rating Inventory of Executive Function, and Conners 3. Disease-related characteristics were extracted from health records.

**Results:**

The K-SCT demonstrated excellent internal consistency (α = 0.92) and test-retest reliability (*r* = 0.82, *p* < 0.001). K-SCT scores were correlated with ADHD-Inattention (*r* = 0.64, *p* < 0.001) and ADHD-Hyperactive/Impulsive (*r* = 0.46, *p* < 0.001) scores, as well as functional outcomes, including learning problems (*r* = 0.69, *p* < 0.001). In multivariate analyses controlling for ADHD symptoms, SCT accounted for unique variance in learning (*b* = 9.67, *p* < 0.01) and executive functioning (*b* = 5.93, *p* < 0.01). Nearly all participants (93%) with elevated levels of co-occurring SCT and ADHD-Inattention symptoms had significant learning problems.

**Conclusion:**

The K-SCT is a reliable and valid measure of SCT in SCD. SCT symptoms are associated with learning difficulties even after controlling for ADHD symptoms. Further research is needed to understand the biopsychosocial factors that lead to SCT symptoms in SCD and examine long-term implications of SCT.

## Introduction

Sickle cell disease (SCD) is an inherited autosomal recessive disorder characterized by a genetic mutation that produces sickle β-globin, causing hemoglobin molecules to polymerize when deoxygenated and leading to hemolysis, inflammation, and vaso-occlusion (1). Millions of people live with SCD globally, primarily in sub-Saharan Africa and Southeast Asian countries, though the exact prevalence is unknown, due in part to the paucity of newborn screening programs (2, 3). SCD is the most common genetic disorder in the United States, affecting nearly 100,000 people, of whom 90% identify as Black (4). Early concerns of SCD center on an increased risk for life-threatening infection and although pain is often described as the hallmark symptom, SCD has the potential to cause myriad acute complications and chronic organ damage (1). The pathophysiology underlying clinical manifestations of SCD is complex and influenced by environmental factors; consequently, there is notable variability in symptom presentation and course (5, 6).

Neurocognitive and neurodevelopmental deficits are a common complication of SCD. Slowed processing speed, attention difficulties, and executive dysfunction are often identified as salient neurocognitive symptoms beginning in the preschool period, often continuing into adulthood (7). Neurodevelopmental syndromes have also been linked to disease severity in SCD with as many as half of all preschool-age children showing evidence of developmental delay with elevated rates of neurodevelopmental issues persisting into adolescence (8–11).

A key disease-specific contributor to these deficits are cerebrovascular complications that interfere with oxygen delivery to the brain. Cerebrovascular effects of SCD can begin in the preschool period and increase steadily in prevalence into adulthood with half or more of all adult patients showing cerebrovascular disease (7, 8, 12). These disruptions to oxygen delivery are believed to underlie the development of silent cerebral infarction, the most common form of acquired brain injury documented in SCD, and changes in white matter tissue that are linked to cognitive deficits (13–15). In contrast to cognitive deficits demonstrated on formal cognitive testing, neurodevelopmental syndromes are based largely on behavioral symptoms and have received less attention as indicators of neurologic sequalae of SCD. The purpose of the present study is to investigate distinct patterns of neurodevelopmental symptoms that may result from the heterogeneity in brain impacts observed in SCD.

Although there are common underlying cerebrovascular mechanisms that affect brain health in SCD, less attention has been paid to the variability in impact among individuals. For example, cerebral infarction from overt stroke often occurs in a variety of regions supplied by the anterior and middle cerebral arteries with lesion locations differing across patients (16). Silent cerebral infarction often involves patchy lesions in the border zone regions between these arterial distributions (13, 16). Thus, the brain effects of SCD occur in a wide range of areas that could disrupt functional connections in the frontal, parietal, and temporal lobes as well as subcortical nuclei such as the caudate and putamen. The effects on deep white matter regions surrounding the lateral ventricles (both anteriorly and posteriorly) also mean small differences in the location of lesions could have implications for the white matter pathways affected.

Despite these individual differences in the functional brain systems most affected, there has been a strong tendency to study the cognitive and behavioral effects of the disease in a unidimensional manner, such as identifying the most common neurocognitive or behavioral sequalae averaged across a group. This approach is an important starting point to understand the most salient and common deficits in a condition but can lead to an underappreciation of heterogeneity. In the present study, we use recent conceptualizations of dimensions of attention-deficit/hyperactivity disorder (ADHD) as a framework for understanding different presentations of neurodevelopmental effects of SCD. More specifically, we examined the extent of symptoms reflecting inattention, hyperactivity and impulsivity, and sluggish cognitive tempo (SCT) to understand if these represent distinct dimensions of SCD-related behavioral complications potentially stemming from variability in underlying brain effects.

In current diagnostic approaches, there are three subtypes of ADHD which reflect two dimensions of symptoms: inattentive symptoms and hyperactive-impulsive symptoms with the third category representing a combined presentation with both dimensions present (17). The predominantly inattentive subtype of ADHD (ADHD-I) has typically been noted as the most common behavioral phenotype observed in youth with acquired brain insults (18, 19). Between 7 and 25% of youth with SCD have been diagnosed with ADHD; though, reports may underestimate true prevalence due to complexities associated with diagnosing ADHD in SCD and the significant barriers to formal evaluation (9, 20, 21).

The dimension of SCT was originally developed within the literature on ADHD as a possible phenotype that may better identify youth with predominantly inattentive symptoms but was later identified as a distinct phenotype from inattentive symptoms that often co-occurs with ADHD-I (22). SCT as a syndrome is marked by pronounced symptoms of daydreaming, drowsiness, being easily confused, difficulty initiating and sustaining effort, low activity level and passivity as compared with inattentive symptoms, such as a short attention span, inattentiveness to what others say, and distractibility. Since the original development of the SCT concept there has been growing support for SCT as either an additional variant of ADHD that is distinct from the predominantly inattentive subtype or perhaps a separate disorder with high comorbidity with ADHD (22, 23).

Cognitive neuroscience methods have been used to identify likely underlying neural systems for SCT that differ from those observed in ADHD, whereas more traditional clinical neuropsychological tools have typically not identified distinct features of SCT (23). Elevated SCT symptoms have been associated with deficits in arousal (both at rest and the regulation of arousal in response to environmental stimuli) and selective attention (specifically, in the engagement and shifting of attention), implicating the autonomic nervous system, thalamic, and posterior parietal systems that underlie these functions (24–27). This contrasts with studies of classic ADHD presentations, which are noted to show deficits in executive function and reward sensitivity with critical regions for these systems in prefrontal and superior parietal brain regions (23, 27, 28). In addition to having different neurocognitive profiles, SCT symptoms have been noted to confer additional functional impacts on academic performance not captured by classic ADHD symptoms, are more likely to be comorbid with internalizing disorders, and are associated with lower family socioeconomic status (23, 29, 30).

Prior research on SCT has examined this construct within people that have ADHD and in typically developing individuals without major health conditions but no studies, to our knowledge, have described SCT in SCD. Therefore, we conducted a study with the following objectives: (1) Determine the reliability and validity of the Kiddie-Sluggish Cognitive Tempo (K-SCT) measure in a sample of youth with SCD; (2) Describe biopsychosocial correlates of SCT symptoms in pediatric SCD; and (3) Examine the unique contribution of SCT symptoms to functional difficulties (e.g., learning problems, mood concerns) and the synergistic influence of SCT and ADHD symptoms on functional outcomes in SCD. We hypothesized that the K-SCT would demonstrate acceptable reliability and validity. It was expected that participants with lower socioeconomic status (e.g., lower parent educational attainment, lower family income) and greater SCD-related neurological risk (e.g., HbSS or HbSβ^0^ thalassemia genotypes, overt stroke, silent cerebral infarcts) would be rated as having more SCT symptoms. We also hypothesized that higher SCT symptoms would be significantly correlated with greater functional impairment (e.g., learning problems, mood concerns) and that SCT symptoms would uniquely account for variance in parent-reported learning problems beyond the influence of inattentive ADHD symptoms. Also consistent with prior studies, we expected that participants with both high SCT symptoms and high inattentive ADHD symptoms would exhibit the greatest degree of functional impairment (23, 29, 30).

## Materials and Methods

### Participants

Children and adolescents with SCD and their caregivers were recruited to participate in a larger clinical trial evaluating a computerized cognitive rehabilitation intervention. Patients were deemed eligible if they were between ages 7–16 years, were fluent in English, were accompanied by a parent or legal guardian, and had reliable access to electricity so they would be able to charge a mobile device at home if they screened into the intervention phase of the trial. Exclusion criteria included having a physical limitation that interfered with computer use (including severe intellectual disability) and recently starting a stimulant medication (<30 days). If patients had recently started a stimulant, they were still invited to enroll in the study after taking the medication for at least 30 days, at which point they would meet eligibility criteria.

### Procedures

Study procedures were reviewed and approved by the Institutional Review Board prior to data collection. Eligible patients were approached during routine hematology clinic visits and recruited to participate. After obtaining informed consent, participants completed a brief neurocognitive screening assessment to identify participants with working memory difficulties who would be eligible to be randomized to either a digital cognitive training intervention arm or an inactive waitlist arm. Data for the present analyses were obtained from all participants at baseline regardless of whether they exhibited working memory difficulties. Data were also extracted from participants in the waitlist arm 5–8 weeks after the baseline assessment, though these data were only used to examine test-retest reliability of the SCT measure. The current study focused on caregiver ratings of behavior and learning completed as part of a baseline assessment. Participants were provided with a $20 gift card and either parking validation or a $10 public transportation pass following each assessment.

### Measures

#### Socio-Demographic, Disease, and Treatment Characteristics

Primary caregivers reported on socio-demographic characteristics including participant age and sex, annual household gross income, and caregiver educational attainment. A medical chart review was conducted to determine SCD genotype (HbSS/HbSβ^0^ thalassemia or HbSC/HbSβ^+^ thalassemia), current treatments (e.g., hydroxyurea, chronic blood transfusion therapy), hemoglobin, and stroke history.

#### Sluggish Cognitive Tempo

The Kiddie-Sluggish Cognitive Tempo measure was completed by caregivers to report on symptoms of SCT (31). This 15-item measure produces a total score and three subscales that aim to quantify: Daydreaming (6 items), Working Memory Slips (5 items), and Sleepy/Tired (4 items). Caregivers answer each item by rating how often their child exhibits certain behaviors as Never or Rarely, Sometimes, Often, or Very Often. The 15-item version of the K-SCT was reduced from a larger item pool based on an exploratory factor analysis that demonstrated these items had high loadings on primary factors of SCT and low cross-loading on measures of ADHD-I symptoms. The K-SCT has been previously shown to have good psychometric properties including strong reliability and validity among children with ADHD (31).

#### Related Constructs and Functional Outcomes

The Conners 3rd Edition Parent-report (Conners 3) was used to measure ADHD symptoms and associated cognitive and behavioral problems (32). Caregivers completed the 99-item Conners 3 by reporting observations of cognitive and behavioral symptoms using a Likert-type scale with four options: 0 (not at all), 1 (just a little true), 2 (pretty much true), and 3 (very much true). The measure consists of several subscales but for the focus of the current study, analyses focused on the following domains: Executive Functioning, Learning Problems, Aggression, Peer Relations, ADHD Predominantly Hyperactive/Impulsive, and ADHD Predominantly Inattentive. The Conners 3 has good internal consistency, with a Cronbach's alpha of 0.91. Previous reports suggest there is no evidence of race or sex bias, and the demographics of the standardization sample reflected the demographic makeup of the 2,000 U.S. Census (33).

The Behavior Rating Inventory of Executive Function (BRIEF) was also administered to assesses caregiver observations of functional impairment across metacognitive and behavioral regulation domains (34). The 86-item BRIEF asks caregivers to choose one of three options (never, sometimes, often) in response to the listed statements. Two primary index scores are produced: The Metacognition Index (MI) and the Behavioral Regulation Index (BRI). The MI score reflects abilities to monitor behavior and performance, organize materials, plan and organize tasks, engage working memory, and initiate tasks. The BRI score represents functioning related to inhibiting behavior, shifting tasks, and controlling emotions. The BRIEF has good internal consistency, with alphas ranging from 0.80 to 0.89. Demographic characteristics of the standardization sample reflected the census distribution, and analyses found that race and ethnicity do not significantly affect BRIEF scores (34).

### Data Analysis

Descriptive analyses were conducted to examine socio-demographic and medical characteristics of the study sample. Unless otherwise noted, data used in analyses were collected at baseline. Cronbach's alpha was calculated to evaluate internal consistency of the K-SCT total score as well as each of the subscales. Paired samples correlations were used to determine test-retest reliability on the K-SCT total score from baseline to 5 to 8-week follow-up. In order to evaluate for convergent evidence of the K-SCT, we examined Pearson correlation coefficients among the K-SCT total score and the Conners 3 ADHD Primarily Inattentive symptom scale and the Conners 3 ADHD Primarily Hyperactive/Impulsive symptom scale. A similar procedure using Pearson's *r* was followed to evaluate test-criterion evidence between K-SCT scores and concurrently measured functional outcomes (e.g., metacognitive functioning, behavioral regulation, learning problems, aggression, and peer relations). Analyses to determine associations between the K-SCT and socio-demographic and disease-related risk factors varied according to the risk variable type (e.g., Pearson's *r* for continuous variables, independent samples *t*-tests for dichotomous variables).

Linear regression analyses were conducted to assess the variance in functional outcomes attributable to SCT after controlling for the influence of ADHD-I symptoms and conceptually relevant covariates. To explore the implications of having a single elevation of domain symptoms (i.e., either high SCT or ADHD-I) compared to a mixed presentation of symptoms (i.e., high SCT *and* ADHD-I), variables were transformed to signal the presence or absence of significant symptoms. Specifically, K-SCT scores were dichotomized at the 75th percentile, as no established cutoffs exist, and Conners ADHD-I scores were dichotomized at a *T* score of 65 (i.e., ≥1 *SD* above the mean). Participants were assigned to one grouping based on the presence or absence of elevated ADHD-I and SCT scores: (1) normal ADHD-I and SCT symptoms; (2) elevated ADHD-I with normal SCT symptoms; (3) normal ADHD-I symptoms with elevated SCT symptoms; and (4) elevated ADHD-I and SCT symptoms.

## Results

Eighty-five caregivers completed the K-SCT at baseline to report on SCT symptoms in their children. Descriptive results for the sample are presented in Table 1. Five caregivers elected not to report gross family income; these cases were excluded from regression analyses where income was used as a covariate. A review of medical records found that a clinical MRI had been obtained for 53 participants (62%) an average of 1.25 years (*SD* = 1.73) prior to study enrollment. The K-SCT exhibited excellent internal consistency, with a Cronbach's alpha of 0.92 for the 15-item total score. Internal consistency ranged from good to excellent for the three subscales, with a Cronbach's alpha of 0.94 for the Daydreaming subscale, 0.87 for the Working Memory Slips subscale, and.81 for the Sleepy/Tired subscale. Test-retest statistics comparing baseline to 5 to 8-week follow-up (*n* = 17) were also acceptable, as paired samples correlations were 0.82 (*p* < 0.001) for the total score, 0.69 (*p* = 0.002) for the Daydreaming subscale, 0.82 (*p* < 0.001) for Working Memory Slips subscale, and 0.77 (*p* < 0.001) for the Sleepy/Tired subscale.

**Table 1:** Descriptive characteristics of the study sample.

	***n* (%)**	**Range**	**Mean (SD)**
**Socio-demographic variables**			
Age		7–16	10.45 (2.97)
Sex (female)	49 (58%)		
Caregiver education (no college degree)	47 (55%)		
Annual family income (<$50,000)	42 (53%)		
**Medical variables**			
Genotype (HbSS/HbSβ^0^ thalassemia)	64 (75%)		
Taking hydroxyurea	46 (54%)		
Chronic blood transfusion therapy	23 (27%)		
Hemoglobin		6.90–14.20	9.40 (1.46)
Overt stroke	7 (8%)		
MRI documented in medical record	53 (62%)		
MRI-confirmed silent infarct	18 (34%)		
**Caregiver-reported behavioral functioning**			
K-SCT total		0–2.60	0.60 (0.50)
K-SCT daydreaming		0–3.00	0.51 (0.65)
K-SCT working memory slips		0–2.60	0.60 (0.56)
K-SCT sleepy/tired		0–2.75	0.74 (0.61)
Conners-3 ADHD primarily inattentive		36–90	57.64 (14.52)
Conners-3 ADHD primarily hyperactive/impulsive		38–90	52.22 (10.94)

*n = 85. K-SCT possible scores range from 0 to 3. Higher K-SCT scores reflect greater sluggish cognitive tempo symptoms. Conners 3 subscale scores represent T scores where the population mean is 50 and the standard deviation is 10. Higher Conners 3 scores reflect greater difficulties*.

Results also provided convergent evidence of the K-SCT in youth with SCD (see Table 2). The baseline K-SCT Total score was significantly correlated with the Conners 3 ADHD Primarily Inattentive Presentation domain score (*r* = 0.64, *p* < 0.001) and the ADHD Primarily Hyperactive/Impulsive Presentation domain score (*r* = 0.46, *p* < 0.001), such that participants who were rated has having more SCT symptoms were also described as having more ADHD Inattentive and ADHD Hyperactive/Impulsive symptoms.

**Table 2:** Correlations between Kiddie-Sluggish Cognitive Tempo scores, related constructs, and socio-demographic and disease characteristics.

	**K-SCT total score**	**Conners-3 ADHD inattentive presentation**	**Conners-3 ADHD hyperactive/impulsive presentation**
**Socio-demographics**			
Age	−0.04	0.06	0.10
Sex	0.01	−0.06	−0.19
Parent education	0.04	0.12	0.05
Family income	−0.03	0.10	0.02
**Medical variables**			
HbSS or HbSβ^0^ thalassemia	−0.19	−0.29^*^	−0.21
Overt stroke	−0.17	−0.08	0.05
Silent infarct	−0.03	−0.08	0.06
Hemoglobin	0.02	0.11	0.08
**Kiddie-Sluggish Cognitive Tempo**			
K-SCT total	–	0.64^**^	0.46^**^
K-SCT daydreaming	0.90^**^	0.58^**^	0.47^**^
K-SCT working memory slips	0.87^**^	0.62^**^	0.44^**^
K-SCT sleepy/tired	0.64^**^	0.32^**^	0.16
**Conners 3**			
ADHD inattentive	0.64^**^	–	0.52^**^
ADHD hyperactive/impulsive	0.46^**^	0.52^**^	–
Learning problems	0.69^**^	0.79^**^	0.42^**^
Executive functioning	0.55^**^	0.92^**^	0.39^**^
Defiance/aggression	0.23^*^	0.31^**^	0.53^**^
Peer relations	0.23^*^	0.18	0.25^*^
**BRIEF**			
Metacognition index (MI)	0.66^**^	0.83^**^	0.51^**^
Behavioral regulation index (BRI)	0.47^**^	0.51^**^	0.71^**^

*n = 85.*

** *Indicates p < 0.01.*

* *Indicates p < 0.05. “Silent infarct” reflects only silent infarcts confirmed by MRI. A chart review found a clinical MRI had been completed for 53 participants*.

SCT was unrelated to parent educational attainment and family income. Also contrary to hypotheses, SCT did not vary significantly by genotype, hemoglobin, or overt stroke. Although SCT also did not vary by silent infarct history, the sample size for this analysis was reduced as the variable was restricted to only those cases where a clinical MRI had been previously conducted (*n* = 53; 62%), of which 34% (*n* = 18) revealed evidence of silent infarct. Results supported hypotheses regarding the associations between SCT and functional outcomes. Greater SCT symptoms as measured by the K-SCT total score were significantly associated with greater caregiver-rated learning problems, metacognitive and behavior regulation difficulties, defiance/aggression, and peer problems (Table 2).

Based on prior literature suggesting conceptual overlap between SCT and ADHD Inattentive symptoms, we examined whether there was a unique influence of SCT on caregiver-rated learning problems, metacognitive functioning, and behavior regulation when controlling for ADHD Inattentive symptoms (Table 3). Linear regressions demonstrated that models incorporating socio-demographic variables, SCD characteristics, ADHD Inattentive and ADHD Hyperactive/Impulsive symptoms, and SCT symptoms as independent variables significantly predicted caregiver-rated learning problems (*R*^2^ = 0.75, *p* < 0.001), metacognitive functioning (*R*^2^ = 0.75, *p* < 0.001), and behavior regulation (*R*^2^ = 0.52, *p* < 0.001). Even after controlling for ADHD symptoms, higher SCT symptoms significantly predicted learning problems (β = 0.35, *p* < 0.001). For every 1-point increase in the K-SCT total score, there was a T-score increase of 9.67 on the Conners 3 Learning Problems subscale. Similarly, after controlling for ADHD symptoms, greater SCT symptoms predicted more difficulties with metacognitive functioning (β = 0.25, *p* < 0.01). Each 1-point increase on the K-SCT Total score was associated with a T-score increase of 5.93 on the BRIEF MI. SCT did not significantly predict caregiver-rated behavior regulation.

**Table 3:** Multiple regression models predicting functional outcomes.

	**b**	**SE**	**β**	***p***	***R* ^2^**
**Dependent variable: Conners-3 learning problems**					
(Constant)	19.33	7.65			0.75^**^
Age	0.48	0.30	0.10	0.11	
Severe SCD genotype	0.63	2.25	0.02	0.78	
Stroke or silent infarct	0.82	2.00	0.03	0.68	
Parent education	−1.01	0.78	−0.09	0.20	
Family income	−0.38	0.51	−0.06	0.45	
Conners-3 ADHD inattentive	0.58^**^	0.08	0.59	<0.01	
Conners-3 ADHD hyperactive/impulsive	0.04	0.10	0.03	0.72	
K-SCT total	9.67^**^	2.23	0.35	<0.01	
**Dependent variable: BRIEF metacognition index**					
(Constant)	14.30	6.07			0.75^**^
Age	0.66^*^	0.25	0.16	0.01	
Severe SCD genotype	1.32	1.88	0.05	0.49	
Stroke or silent infarct	−0.37	1.67	−0.01	0.83	
Parent education	−0.65	0.65	−0.07	0.32	
Family income	0.80	0.42	0.14	0.06	
Conners-3 ADHD inattentive	0.52^**^	0.07	0.63	<0.01	
Conners-3 ADHD hyperactive/impulsive	0.06	0.08	0.05	0.45	
K-SCT total	5.93^**^	1.86	0.25	<0.01	
**Dependent variable: BRIEF behavioral regulation index**					
(Constant)	6.93	7.51			0.52^**^
Age	0.57	0.61	0.16	0.07	
Severe SCD genotype	3.31	2.33	0.14	0.16	
Stroke or silent infarct	−0.34	2.07	−0.02	0.87	
Parent education	0.15	0.80	0.02	0.86	
Family income	−0.35	0.52	−0.07	0.51	
Conners-3 ADHD Inattentive	0.10	0.08	0.14	0.23	
Conners-3 ADHD Hyperactive/Impulsive	0.57^**^	0.10	0.55	<0.01	
K-SCT Total	3.03	2.30	0.14	0.19	

* *Indicates p < 0.05;*

** *Indicates p < 0.01*.

**Figure 1:**
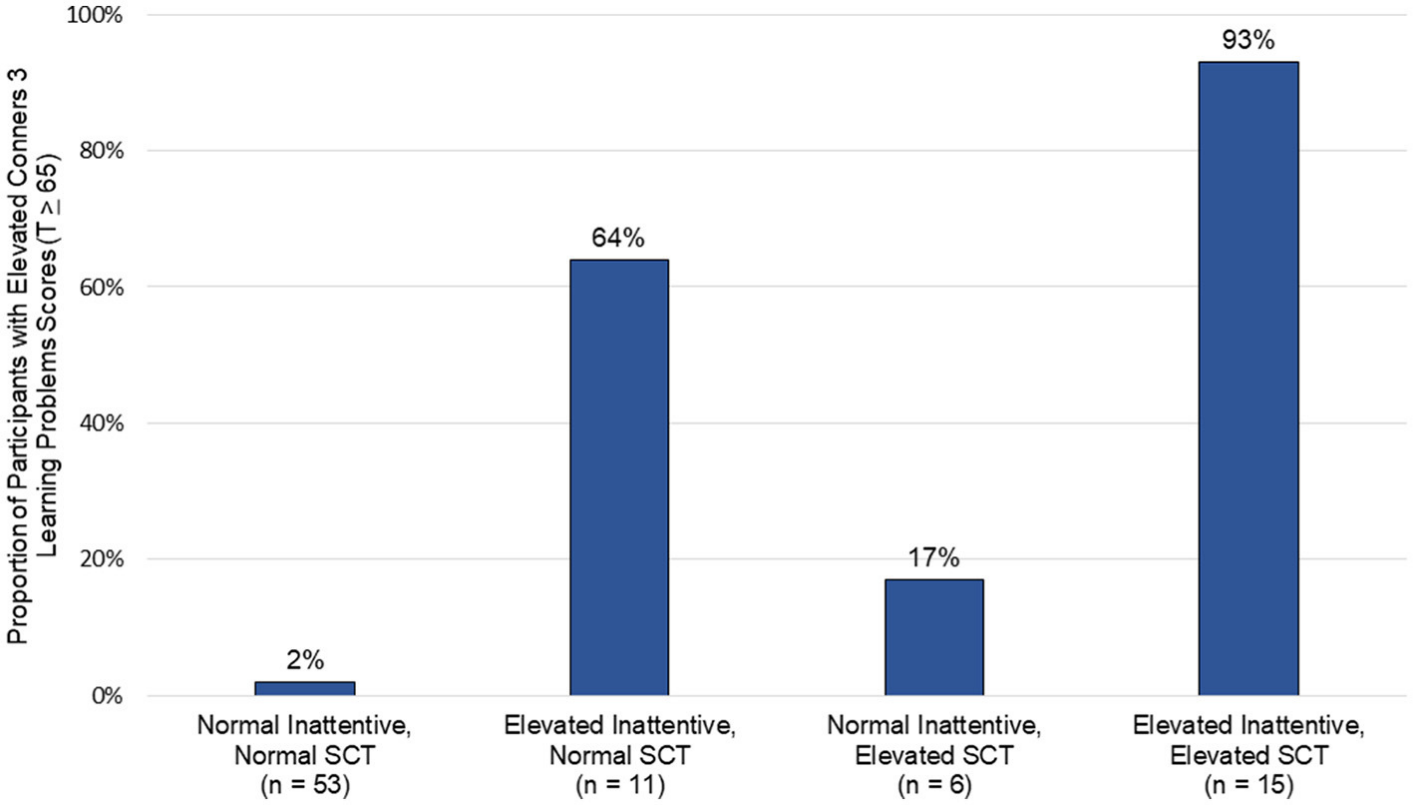
Clusters of inattentive and sluggish cognitive tempo symptoms and their association with learning problems.

Participants were grouped based on the presence of significant SCT symptoms (>75th percentile; *n* = 21; 25%) and ADHD-I symptoms (T score > 65; *n* = 26; 31%) to better characterize the functional effect of SCT, ADHD-I, and the combination of SCT and ADHD-I symptoms on learning problems (see Figure 1). Conners 3 Learning Problems subscale means and standard deviations are available in Supplementary Table 1. Most participants were categorized as having normal levels of both ADHD-I and SCT symptoms (*n* = 53; 62%). The next largest group had both clinically elevated ADHD-I and SCT symptoms (*n* = 15; 18%), followed by elevated ADHD-I symptoms with normal SCT symptoms (*n* = 11; 13%), and normal ADHD-I symptoms with elevated SCT symptoms (*n* = 6; 7%). A majority of participants with clinically elevated ADHD-I symptoms were rated as having significant learning problems, regardless of their degree of SCT symptoms (21 of 26 participants; 81%). Similarly, most participants with elevated SCT symptoms had significant learning problems, regardless of ADHD-I symptoms (15 of 21 participants; 71%). While most participants with elevated ADHD-I symptoms and normal SCT symptoms were rated as having significant learning problems (7 of 11 participants; 64%), nearly all of those with both elevated ADHD-I and SCT symptoms had learning problems (14 of 15 participants; 93%).

## Discussion

The current study characterized SCT symptoms in children and adolescents with SCD by reporting on the reliability and validity of a parent proxy-report measure of SCT symptoms, examining associations between SCT and socio-demographic and disease-related risk factors, and describing the effect of SCT symptoms and combined ADHD-I and SCT symptoms on functional outcomes. Reliability and validity analyses supported the K-SCT as a reliable measurement tool with evidence of convergent and test-criterion validity. Findings also revealed that, contrary to hypotheses based on the broader ADHD literature, we were not able to identify risk factors for SCT symptoms among youth with SCD: SCT symptoms did not vary by any socio-demographic factors or SCD-related risk variables that were measured. Data did, however, support the clinical significance of SCT symptoms: Higher SCT symptoms were associated with functional outcomes even after controlling for ADHD-I symptoms. In addition, the combined presentation of SCT and ADHD-I symptoms conferred significant risk for learning problems.

In order for novel phenomena to be studied, rigorous methods of measurement are needed. The K-SCT has been used to study SCT in typically developing children and those with other neurodevelopmental diagnoses but to our knowledge, its psychometric properties had not been evaluated in SCD. Results supported the 15-item K-SCT as a reliable and valid instrument for assessing SCT symptoms in children ages 7–16 with SCD. Data suggested that the K-SCT has excellent internal consistency and produces reliable scores across a 5 to 8-week period. Moreover, results provided convergent evidence for the K-SCT as the K-SCT total score was significantly correlated with the Conners 3 ADHD Primarily Inattentive and ADHD Primarily Hyperactive/Impulsive subscales. Many associations followed expected patterns, with K-SCT scores correlating stronger with symptoms of inattention than symptoms hyperactivity and impulsivity. These findings provide initial support for the reliability and validity of the K-SCT and suggest that it could be used to further investigate SCT in pediatric SCD.

Based on previous studies suggesting associations between socio-demographic risk factors and neurocognitive outcomes in typically developing children and in youth with SCD, we expected that demographic characteristics and measures of socioeconomic status would be related to SCT symptoms. However, K-SCT scores did not vary by age, sex, parental education, or family income. These expectations were derived primarily from prior studies of youth with ADHD, and it may be that associations do not generalize across all populations at risk for SCT symptoms, particularly among more diverse groups exposed to a multitude of neurologic risks. It is also possible that community-level factors unique to the region where patients were recruited (e.g., proximity to a large children's hospital with a comprehensive SCD care clinic and dedicated social workers, high quality schools and other community resources, access to early intervention services) helped buffer the effect of broad proxy measures of socioeconomic status.

Our findings may also reflect nuances of measuring socioeconomic risk and associated outcomes. Prior studies point to variability in relationships between socioeconomic variables and neurocognitive outcomes that depend in part on the domain examined and how socioeconomic risk is operationalized. Strong evidence supports a connection between socioeconomic status (parent educational attainment, in particular) and global intellectual functioning in SCD (35), whereas others have reported no association between socioeconomic status and certain neurocognitive abilities (36). Researchers have been attuned to the complexity of measuring socioeconomic and social-environmental risk factors, recognizing that parent educational attainment and income are important predictors that lead to downstream risks (e.g., parenting stress) known to more directly influence neurodevelopmental and behavioral outcomes.

Variability in defining and measuring risk inevitably leads to conflicting findings in the literature, especially when studying heterogenous groups such as youth with SCD. For instance, Yarboi and colleagues found that maternal experiences of financial stress predicted performance on measures of verbal intelligence but not visual-spatial functioning and non-verbal reasoning in school-age children with SCD (37). Similarly, Bills et al. reported that, in a sample of young children with SCD, traditional measures of socioeconomic status were not uniformly correlated with neurocognitive performance (36). Moreover, they found that parent and family functioning (e.g., positive parent-child interactions, caregiver warmth and support) predicted phonological processing and ADHD symptoms over and above socioeconomic status (36). Future research should incorporate an intentional and multimodal approach to measuring social-environmental risk factors and evaluate effects on neurodevelopmental outcomes prospectively to better explicate these complicated relationships.

Results also suggested that disease-related variables were not associated with SCT. A limitation of this study was that neuroimaging data were not consistently collected across participants. Prior research also suggests a more specific pattern of neurocognitive deficits (e.g., specific to arousal regulation) may be important for SCT symptoms, rather than simply examining the presence/absence of neurologic injury. The available neuroimaging data identified relatively few participants with silent infarcts and overt strokes in our sample and data regarding infarct location and size that could have helped to clarify potential associations between neural injury and SCT were not available. Thus, there were multiple methodological factors that could account for these null findings. Future research should consider including more refined measures of structural brain effects and cognitive neuroscience methods that could identify those children with specific neurocognitive deficits associated with SCT in prior research. It may also be useful for future studies to incorporate prospective neuroimaging, which would enable characterization of pathways that give rise to distinct neurocognitive phenotypes over time and could determine effects of disease-modifying therapies on the prevention or slowing of neurocognitive decline.

The most salient aspect of the current findings was the influence of SCT on functional outcomes. High SCT was associated with more problems with learning, metacognitive functioning, executive abilities, behavioral regulation, defiance and aggression, and peer relationships. These findings suggest that SCT symptoms significantly contribute to learning problems and negatively influence cognitive, behavioral, and social outcomes in youth with SCD. Furthermore, regression models controlling for ADHD-I symptoms appeared to suggest that SCT symptoms represent a unique risk to functional outcomes including learning problems and metacognitive functioning difficulties.

Additional evidence is needed to more fully understand the extent to which ADHD-I and SCT represent overlapping or distinct neurodevelopmental presentations. The influence of SCT was especially prominent among children who had elevated ADHD-I symptoms, as they were rated as having the greatest difficulties with learning, metacognition, and behavioral regulation. Ninety-three percent of participants with this combined presentation of high ADHD-I and SCT symptoms demonstrated clinically significant learning problems. Therefore, elevated SCT symptoms appear to be not only independently related to real-world neurocognitive, behavioral, and social challenges, but also contribute to a synergistic effect on functional outcomes when occurring in the setting of significant ADHD-I symptoms.

This finding has implications for the treatment of ADHD in SCD and efforts to mitigate behavioral and learning difficulties, as SCT has been shown to be associated with non-response to stimulant medication for children with ADHD (38). Although there is no indication that evidence-based treatments for ADHD will not be similarly effective in youth with SCD with ADHD, studies have also demonstrated the availability of safe and efficacious treatments for attention and working memory in SCD (39, 40). Optimizing available treatments for neurodevelopmental syndromes in SCD is a critical need, given the reportedly low rates of engagement in behavioral (~50%) and pharmacologic treatments (21–63%) for ADHD (9, 21). Tailoring interventions to specific neurodevelopmental phenotypes (e.g., ADHD-I and SCT symptoms) has the potential to enhance efficacy and could affect interest in treatment; though, facilitators and barriers to accessing care need further investigation.

SCT is a neurodevelopmental syndrome that has been recently described and may be underappreciated due to confusing its symptoms with those of ADHD-I. Additional research on SCT is needed to advance our understanding of the ways SCD negatively affects everyday functioning, including the high rate of learning difficulties observed in SCD. SCT symptoms may be particularly important for their negative synergy with ADHD-I symptoms in producing learning difficulties; however, larger samples are needed to conduct analyses that can better disentangle complex cognitive phenotypes and overlapping symptom clusters (e.g., latent profile analysis). More research is also needed to understand the long-term functional implications of SCT symptoms and the specific etiological factors in SCD that may increase risk for SCT. In particular, there are specific functional neural systems that have been found to be disrupted among children with SCT symptoms and future research should consider examining the integrity of these neurocognitive systems in relation to SCT symptoms in SCD to better identify how to prevent these difficulties.

## Data Availability Statement

The raw data supporting the conclusions of this article will be made available by the authors, without undue reservation.

## Ethics Statement

The studies involving human participants were reviewed and approved by Children's National Institutional Review Board. Written informed consent to participate in this study was provided by the participants' legal guardian/next of kin.

## Author Contributions

SH, KH, and JS contributed to conception and design of the study. SH performed the statistical analysis. SH and JS wrote the first draft of the manuscript. SF and KH wrote sections of the manuscript. All authors contributed to manuscript revision, read, and approved the submitted version.

## Funding

This work was supported by the Doris Duke Charitable Foundation (Grant #2013141 to SH and KH) and the National Institutes of Health (Grant #K23HL14166-01A1 to SH).

## Conflict of Interest

The authors declare that the research was conducted in the absence of any commercial or financial relationships that could be construed as a potential conflict of interest.

## Publisher's Note

All claims expressed in this article are solely those of the authors and do not necessarily represent those of their affiliated organizations, or those of the publisher, the editors and the reviewers. Any product that may be evaluated in this article, or claim that may be made by its manufacturer, is not guaranteed or endorsed by the publisher.
