# Supplementary material for: Sluggish Cognitive Tempo in Pediatric Sickle Cell Disease
Source: Front Neurol. 2022 Jul 7;13:867437. doi: 10.3389/fneur.2022.867437 (PMC9301245; doi:10.3389/fneur.2022.867437)
Supplement: Supplementary file 1 [file Data_Sheet_1.PDF]

Supplemental Table I. Mean Scores on Functional Outcomes Across ADHD-Inattention and Sluggish Cognitive Tempo Symptom Clusters

	Conners 3 Learning Problems	BRIEF Metacognition Index	BRIEF Behavioral Regulation Index
Normal ADHD-I and Normal SCT	50.79 (8.21)	50.89 (8.55)	48.25 (7.85)
Elevated ADHD-I and Normal SCT	66.09 (8.88)	67.00 (8.25)	49.09 (6.33)
Normal ADHD-I and Elevated SCT	61.50 (11.08)	62.33 (3.50)	55.83 (11.20)
Elevated ADHD-I and Elevated SCT	77.80 (10.83)	72.47 (6.05)	65.00 (13.84)

Note. N = 85. Scores reported are T scores with standardized means of 50 and standard deviations of 10. Clinically elevated scores are $\geq$ 65. "ADHD-I" refers to the Conners-3 ADHD Primarily Inattentive domain. "SCT" refers to the Kiddie-Sluggish Cognitive Tempo total score. Cutoff for elevated ADHD-I scores is a T score of 65. Cutoff for elevated SCT scores is at the 75th percentile.
